# An Overview of the International Literature on Health-Related Quality of Life, Mental Health and Psychosocial Issues in People with Cancer

**DOI:** 10.2174/1745017902117010253

**Published:** 2021-12-31

**Authors:** Jutta Lindert, Federica Sancassiani, Elena Massa, Antonio Egidio Nardi

**Affiliations:** 1 University of Applied Sciences Emden/Leer, Emden, Germany; WRSC, Brandeis University, Waltham, United States; 2 Department of Medical Sciences and Public Health, University of Cagliari, Cagliari, Italy; 3 Institute of Psychiatry, Federal University of Rio de Janeiro, Rio de Janeiro, Brazil

**Keywords:** Cancer, HRQol, Mental illnesses, Bipolar disorders, Mental health, Depression

## Abstract

**Background:**

Cancer is one of the most important leading causes of death worldwide. Early detection, screening and diagnosis have been demonstrated to significantly improve patients’ survival rates and increase awareness of the benefit of prompt therapies and healthy lifestyles. In this context, Health-related Quality of Life (HRQoL) and several psychosocial difficulties are of relevance as prognostic factors for the trajectory of the diseases of people living with cancer.

**Methods:**

This Special Issue aims to present a set of systematic reviews and research studies focusing on several psychosocial aspects in people suffering from hematologic and solid cancer.

**Results:**

Three systematic reviews regard HRQoL, the quality of patient-physician communication, depression and other stress-related difficulties, respectively. One review pointed out the difficulties in diagnosing depression in the elderly with solid cancer; another one regards the risk of cancer in severe mental illnesses, such as schizophrenia, bipolar disorders, and severe depressive disorders. One additional review regards HRQoL in people with cancer in the present era of COVID-19 pandemic. Furthermore, some research studies pointed out the usefulness of a validated instrument to assess satisfaction with care in the oncology field, as well as of the self-reinforcing feedback loop to improve fatigue, insomnia and depression in people with cancer. Other two research studies evaluate, respectively, the attributable burden in worsening HRQoL in people suffering both from cancer and depression and the Type D personality as a risk factor for stress-related difficulties in women with breast cancer.

**Conclusion:**

This Special Issue is a contribution to enhance future research mainly about such interventions useful to assess and improve HRQoL and overall well-being in people with cancer.

## 1 BACKGROUND

The World Health Organization (WHO) pointed out that 9.6 million people worldwide died from cancer in 2018 [1], such that cancer is a major public health issue [2].

When a person receives a diagnosis of cancer, his/her psychosocial and physical wellbeing starts to be compromised, due to the illness and the adaptation process that it requires, as well as to the long-term and often painful treatments [3, 4]. Furthermore, symptoms like fatigue [5] insomnia [6], anxiety and depressed mood [7] often occur in people who are living with cancer, and these clinical conditions could be also associated with personality traits, such as Type D [8, 9]. Type D (distressed) personality refers to the tendency to often experience negative affectivity and social inhibition [10] and, particularly among people with cancer, it seems to increase the risk of impaired Health related Quality of Life (HRQoL) and mental health problems that cannot be explained by socio-demographic or clinical variables, such as the kind of cancer or the cancer’ treatment [8].

As shown by many studies, this high amount of distress has inevitably a strong impact on the HRQoL of people affected by cancer [11-14].

HRQoL is a complex and multidimensional construct that is associated with gender and socioeconomic status [15], but also with illness, illness perception, mental health, satisfaction with leisure time, feelings of belonging, perceived vitality, perceived pain, and quality of sleep [16, 17]. The concept of HRQoL is clearly related also to the definition of bio-psycho-social health proposed by the WHO [18], as “a state of complete physical, mental and social well-being and not merely the absence of disease or infirmity”.

Some research points out that HRQoL may predict survival rates in people with cancer [19-21]. Due to this knowledge, since the 80s, the U.S. Food and Drug Administration (FDA) [22, 23] has recommended, along with traditional clinical outcomes and other patient-reported outcomes (PROs), a reliable assessment of HRQoL also in oncology.

Satisfaction with care is another topic which can be improved in people suffering from cancer [24-27]. Satisfaction with care may partly reflect matching of psychosocial expectations and needs of the patients with cancer, and the health professional capacity for meeting these needs, by empathy and interest for the patients [26]. Another factor that could have an impact on satisfaction with care in people suffering from cancer is the quality of the physician–patient communication [28, 29]. Effective communication facilitates patients’ satisfaction with care including patients living with cancer. Additionally, it might contribute to adherence to treatments, reduce distress and increase HRQoL [30, 31]. Furthermore, effective communication between people with cancer and health professionals is fundamental to optimize counseling interventions to improve healthy lifestyles such as diet and nutrition [32].

Among the several factors that potentially contribute to worse HRQoL, there is especially Major Depressive Disorder (MDD) [33-35]. Compared to the general population, MDD is more common among individuals with cancer, and it is associated with increased mortality rate in this kind of population [7, 36-38].

Several studies pointed out bio-behavioral mechanisms to explain the relation between depression and cancer survival [7, 9, 37-41]. However, depression and psychological distress [42, 43], as well as other mental disorders [44], are poorly recognized in people suffered from cancer, because often they are not routinely screened. This is evident among older people, since it might be that the elderly report depressive symptoms differently than younger people. Additionally, they do not only report symptoms differently but depressive symptoms and clinical signs of cancer or side-effects of cancer’ treatments may overlap [45, 46]. Symptoms such as fatigue and sleep disturbance are frequent in people suffering from depression, cancer or both conditions. Furthermore, suffering from these symptoms contribute to self-reinforce the feedback loop with depressive [47-49] and cancer symptoms and herewith potentially improve both, depressive and cancer symtoms [49].

Conversely, a lower percentage of people suffering from depression or other psychosocial disabilities receive proper treatments for cancer due to poor mental health literacy and stigma, resulting in survival after cancer diagnosis significantly worse than people without this comorbidity [44].

Recently, due to the COVID-19 pandemic, this situation is further increased worldwide, with a significant decrease and delays in cancer treatments and screening [50], as well as in the assessments of HRQoL and the other PROs [51, 52]. In the next years, the delay in cancer care could change the recent decline in the cancer mortality rate that has been observed since 1991 [53]. The consequences could be an increasingly demand of such interventions able not only to improve screening campaigns and treatments for cancer and related comorbidities, but also of those actions and programs to improve HRQoL, other PROs and the overall psychosocial wellbeing of people affected.

## 2 AIMS AND GOALS OF THE THEMATIC ISSUE

This Thematic issue includes a set of systematic reviews and research articles aimed at offering a comprehensive exploration of the existing literature with focus on several topics mainly regarding HRQoL, mental health and psychosocial issues in people with cancer and other diseases.

Particularly, one review focused on RCTs showing the efficacy of several cancer’ treatments at improving HRQoL in people suffering from hematologic cancers, such as myelodysplastic syndrome [21]. An overview collected evidences on the positive impact of good physician-patient communication on adherence to treatment, better decision making and satisfaction with care and less medical malpractice claims in people suffering from chronic myeloid leukemia or myelodysplastic syndrome [28].

Three reviews are focused on depression and other psychosocial disabilities in people with cancer. Particularly, the first one pointed out the difficulties in diagnosing depression in elderly with cancer [46], another one regards the risk of cancer in people suffering from severe mental illnesses [9], the last one points out the relation between cancer-related inflammation an depression [41]. One additional review regard HRQoL in people with cancer in the present era of COVID-19 pandemic [52].

Furthermore, this Special Issue includes some research studies aimed to point out the usefulness of a validated instrument to assess satisfaction with care in oncology and psycho-oncology [24], as well as of the implications of the self-reinforcing feedback loop to improve fatigue, insomnia and depression in people with cancer [49]. Other two studies evaluate, respectively, the attributable burden in worsening HRQoL in people suffering both from cancer and depression [12] and the Type D personality as a risk factor for stress-related difficulties in women with breast cancer [9].

## CONCLUSION

The articles included in this Special Issue are a contribution to address further research and interventions, particularly concerning the ways to improve the overall bio-psycho-social health of people suffering from cancer and other related diseases. Actually, HRQoL and other PROs are considered as core indicators to focus effective interventions to support people with cancer in their coexistence with the illness, to better cope with related symptoms and painful treatments, as well as to promote the best adaptation process implied in their daily lives.

This focus requires a shift from a symptoms-centered perspective to care cancer and its related disturbances to a view in which the subjective perception of people suffering from cancer, the psychological resonances with the illness(es) they suffer from and their decision-making process about cares are considered seriously by health professionals.

From a public health perspective, this calls for a strong effort and committed challenge, especially in this era in which the COVID-19 disease has assaulted all the aspects of all people daily life, particularly that of the most vulnerable populations of people already suffered from severe medical illnesses such as cancer.
